# Richmond Academy of Medicine and Surgery

**Published:** 1896-07

**Authors:** Mark W. Peyser

**Affiliations:** Secretary and Reporter


### Society Reports.

### RICHMOND ACADEMY OF MEDICINE AND SURGERY.

### Regular Meeting, May 26, 1896.

Dr. J. N. Upshur, First Vice-President, in the Chair.

The special order for the evening was the discussion of

### BOVINE TUBERCULOSIS: ITS RELATION TO PUBLIC HEALTH.

By Wm. S. Gordon, M. D., Richmond, Va.,

Professor of Physiology, University College of Medicine.

In laying this subject before you for consideration, I have divided it for clearness and convenience into seven heads.

First.—*Do cows, etc., have tuberculosis?*

There can be no doubt of this in the mind of any person who has attended a post mortem. The question need not be discussed from a local point of view, but from a world-wide one; but being answered so emphatically in the affirmative, it would be hardly worth while to go into it.

Second.—*Is it transmitted to animals?*

Sooner or later animals which are injected with the juice of infected meat have the disease. In only one instance, has an observer reported that he was unable to produce the disease by injection. An Italian experimenter states it cannot be communicated to birds. Sheep and goats, particularly the latter, show well-marked tubercles.

I quote the rules and regulations of the Copenhagen Milk Supply Co. to show how it sought to prevent transmission. Reports show cases of congenital tuberculosis in calves; but if a calf of a tuberculous cow be fed with uninfected milk it may be reared in a healthy condition.

Serum of a tubercular human injected into a guinea-pig, has produced tuberculosis.

Third.—*Can it be detected?*

It is not absolutely determined that tuberculin is an infallible diagnostic agent, but the Massachusetts State Board of Health says that in connection with physical signs, it never fails to diagnose; that implicit confidence may be placed in it, and that the reaction is not always in proportion to the stage of disease. A French observer states that inoculation is the one method capable of giving results free from ambiguity. It is a well known fact that at times patients may be dying of phthisis and yet the sputum does not reveal it.

To show the value of tuberculin, statistics prove that out of fifty-three animals injected, forty-one gave the reaction, and of these, thirty-eight proved to be well-marked cases. Many other instances may be quoted with like results; and taking all things into consideration, we see that tuberculin diagnoses almost infallibly.

FOURTH.—*How are herds infected?*

Dr. Harbough states that intestinal tuberculosis is more dangerous than that of the glands or lungs. The animal has diarrhœa, the discharge dries, mixes with dust, is stirred up, breathed, and taken in with the food. He claims this is the common method of infection.

FIFTH.—*Are tubercle bacilli found in the spermatic fluid and milk?*

Referring to the human being, reports are somewhat contradictory, but it is a fact that seminal fluid may transmit the disease even when there is no lesion of the urinary tract. The bacilli are not always found in milk, but, by means of the centrifuge, they have been demonstrated. Ashmead has shown that kiliing of the tuberculous animals, and keeping children from their milk, has increased the longevity of life, and caused a decrease of tuberculosis.

It is claimed that milk is not only dangerous because of bacilli in it, but, also, because of toxins generated by them. Armstrong holds that milk is more apt to infect than meat. Brush says the chronic forms of tuberculosis are not easily recognized, and that the necessity for more practical knowledge is evident. Euleris thinks it significant that a coincidence exists between tuberculous milk and meat and human phthisis.

SIXTH.—*Do animals infect human beings through infectious milk and flesh?*

Concerning milk, observers quoted and others, say it does. The juice of infected meat, injected, produced the disease as cited in the foregoing. Law, of Cornell, says the cooked meat and milk of infected animals are dangerous because of ptomaines in them; citing cases to that effect. Calves sucking tuberculous cows did not thrive, and in those who survived, calcified tubercles were found. Another observer states there is but little danger, but the animals used in experimentation were those not easily affected. It is the consensus of opinion of the College of Physicians and Surgeons, of Philadelphia, that children are apt to be effected by intestinal tuberculosis when fed with infected milk. Fair says the germs are not killed by boiling, a protective coat of coagulated albumin being formed around them.

SEVENTH.—*Does tuberculin harm healthy cows?*

This is an important question, and not only from a hygienic, but also a monetary point of view. I am not now prepared to discuss it.

DISCUSSION.

DR. PAULUS A. IRVING rose to substantiate in a practical way, the authorities and citations given. There is hardly a doubt that bovine tuberculosis may be transmitted to man. It is the chief cause, especially in children. A practical demonstration by Dr. Niles, State Veterinarian, on the Grant herd, eight miles from the city, was witnessed by him. Out of fifty cattle injected, nine gave the reaction, and these Mr. Grant decided to kill. Post mortem, in every instance, showed marked signs of the disease. Two—one a large, healthy-appearing bull—evidenced tuberculosis not only in the cervical, but in the mesenteric glands and lungs. The remaining seven could be diagnosed almost on inspection. All killed were thoroughbreds.

To test the reliability of tuberculin, a purse was made up by the gentlemen present, and a cow that had been passed as healthy, but which was the worst looking of the lot, was purchased and killed. Not a sign of tuberculosis could be discovered. A calf of four or five months was taken from one of the slaughtered animals and examined, but it presented no appearance of the disease.

DR. JNO. N. UPSHUR thinks it is incumbent on us to allay the panic now in existence in this community regarding tuberculosis. There is no doubt we should have an inspector, but improper food and drink may be taken in and digested, the poisonous principle being killed by the juices. The majority of those who within the past ten years have died in Richmond, have had a minimum amount of milk and meat. He doubts if any physician present has traced a case of consumption to milk or meat. He doubts the contagiousness of tuberculosis, believing its starting point to be in malnutrition of the system, making a fertile soil for the development of the bacilli. There recently appeared a statement that the secretion of bronchitis is antagonistic to the germ. Before the war, one of the most common sights was scrofula in the negro, the older writers asserting that consumption was comparatively rare. Why weren't they affected? Since the war, all conditions surrounding them have changed—malnutrition, overcrowding, etc.—the result being phthisis, which will eventually kill the race.

DR. HUGH M. TAYLOR remarked that if it be necessary to come to some tangible conclusion, it is better for the people to be panicky. The question resolves itself into, "Are you willing to drink the milk or eat the meat of a tuberculous cow? Are we, as doctors, willing to continue delicate children on tuberculous milk, and advise our patients to eat tuberculous meat?" He has been asked for advice, then he tells of dairymen who have clean bills of health, and counsels dealing with them. He is not interested in the delicate points, and he hopes the matter will be settled by agitation, for in this way only may we have proper laws enacted. He has no doubt as to the contagiousness of tuberculosis. Time after time he has seen a healthy wife nurse an infected husband, and *vice versa*, only to follow the deceased to the grave from the same cause.

DR. UPSHUR said he did not wish it to appear that he was opposed to legislation on the subject. He objected to the production of a panic by trying to make the danger seem greater than it really is. Milk and meat is constantly subjected to boiling, and there are vital processes that antagonize the poisonous principle.

DR. J. S. WELLFORD agrees with Dr. Upshur that consumption is not contagious, and he can cite cases to prove it. One man had three wives to die of consumption, but he never had it. He can give instance after instance where members of families with a tendency, have married healthy people and never had it, although nursing tuberculosis. He agrees, too, with Dr. Upshur regarding the harm produced among the laity, by agitation of the subject. He believes tyrotoxicon has killed more infants and children than tuberculosis.

DR. JACOB MICHAUX hasn't the slightest doubt as to the contagiousness of tuberculosis. Like Dr. Taylor, he has seen husbands contract it from their wives and *vice versa*.

Concerning its relation to food every effort should be made to impress on lawmakers the importance of laws to restrict the sale of milk and meat.

DR. GORDON, in closing the discussion, remarked that he differed with Drs. Upshur and Wellford as to the contagiousness and infectiousness of tuberculosis, believing that the disease has been proven to be both contagious and infectious. The preponderance of testimony is on this side of the question, and it is impossible not to accept the numerous and convincing operations and experiments made by the leading medical minds of the world to uphold their position. It is unscientific to argue that the disease is not contagious, because heredity frequently has some influence in its production. It is misleading to state that tuberculosis is not conveyed by milk and meat, because all who drink the milk or eat the meat of tuberculous animals, do not contract the disease. It would be equally as logical to conclude that all the inhabitants of a given section should present manifestations of malarial poisoning because a few persons did not suffer from breathing the infected air. The fact that the bacilli may not be detected in every examination of sputum, does not in any way invalidate the views held of the infectious nature of tuberculosis, whether the bacilli be the cause or the result of the disease. It must be remembered that the bacilli are found in the vast majority of cases—their absence from any given sample of sputum does not imply that they are not present somewhere in the respiratory tract, and that if none at all were found, it would not follow that the specific cause of tuberculosis was the germ *per se*, and that the poison, whatever it is, might not be conveyed by infected milk.

He could not state from personal experience whether tuberculosis ever had a deleterious influence upon healthy cows.

The experiments made by veterinarians, however, go to prove that they are correct in asserting that no harm is done.

The reason why the negro, and especially the mulatto, is more liable to the disease at this time, is because, for many reasons, his system is unable to antagonize the poison, and because there is more of the poison in his surroundings. The practical point to be considered is, how shall we get rid of the exciting cause, which, added to the predisposing causes, lights up the disease? It is often impossible for us to control predisposing causes, but, fortunately, we are often able to remove the exciting factor.

He could not agree with Dr. Wellford that tyrotoxicon kills more infants than milk of tuberculous cows. The facility with which germs are absorbed and multiplied in milk, the demonstrated presence of the tubercle bacilli in milk, its widespread use as a food, the known and proven fact that so many infants and children die of tuberculosis,—all of these facts present us with the strongest circumstantial evidence that could be desired.

DR. GORDON said he did not wish to help in causing a panic, but if people were obliged to get into a panic because we told them simple truths in order to educate them and save their lives and the lives of their offspring, then it might be best to have a panic. The panic would not do us as much harm as tuberculous meat and milk.

As for the dairymen, he was anxious to get some legislation which would be beneficial to them as well as to the community. If possible he would have the owners paid for the condemned animals. The laws ought to bear equally on all; and he was sure our dairymen who wish to know the truth and act upon it for their own sakes as well as for the sakes of their patrons, would be willing and anxious to adopt a measure by which the whole community would be benefitted.

MARK W. PEYSER, M. D.,
Secretary and Reporter.

